# Toxicity associated with capecitabine plus oxaliplatin in colorectal cancer before and after an institutional policy of capecitabine dose reduction

**DOI:** 10.1038/sj.bjc.6605995

**Published:** 2010-11-09

**Authors:** R Baird, A Biondo, V Chhaya, J McLachlan, A Karpathakis, S Rahman, Y Barbachano, D Cunningham, I Chau

**Affiliations:** 1 Department of Medicine, Royal Marsden Hospital, London & Surrey, UK; 2 Department of Clinical Research and Development, Royal Marsden Hospital, Surrey, UK

**Keywords:** colorectal cancer, oxaliplatin, capecitabine

## Abstract

**Background:**

Capecitabine plus oxaliplatin (CAPOX) is an established treatment option in colorectal cancer, but can be associated with severe toxicities.

**Methods:**

Following reporting of severe diarrhoea and dehydration with capecitabine 2000 mg m^–2^ per day plus oxaliplatin every 3 weeks (CAPOX 2000) in 2006, we instituted a policy change to reduce capecitabine dose to 1700 mg m^–2^ per day (CAPOX 1700). We undertook a retrospective analysis comparing toxicities encountered before and after this dose change.

**Results:**

Of the 400 patients treated, no significant differences were seen between the CAPOX 2000 and CAPOX 1700 in grades 3 and 4 diarrhoea (21% *vs* 19% *P*=0.80), stomatitis (0% *vs* 1% *P*=0.50) or grades 2–4 hand foot syndrome (16% *vs* 11% *P*=0.18). Grades 3 and 4 neutropenia (9.5% *vs* 3.5% *P*=0.03) and all grades hyperbilirubinaemia (60% *vs* 40% *P*<0.0001) were significantly reduced with CAPOX 1700. Rates of hospitalisation due to toxicities were not different between two groups (13% *vs* 11% *P*=0.53).

**Conclusions:**

No clinically or statistically significant differences in gastrointestinal toxicities or hospitalisation rate were seen after reducing our routine capecitabine dose from CAPOX 2000 to CAPOX 1700.

Capecitabine plus oxaliplatin (CAPOX) is considered to be a standard treatment option in advanced colorectal cancer (CRC) (Cassidy *et al*, 2008) and as adjuvant therapy in colon cancer (Haller *et al*, 2010). In its standard dose schedule of capecitabine 2000 mg m^–2^ per day (days 1–14) and oxaliplatin 130 mg m^–2^ (day 1) every 3 weeks (CAPOX 2000), the reported rate of grades 3 and 4 diarrhoea rate was about 20% (Schmoll *et al*, 2007; Cassidy *et al*, 2008; Rothenberg *et al*, 2008; Haller *et al*, 2010). This severe diarrhoea rate further increased when CAPOX 2000 was combined with cetuximab (Adams *et al*, 2009). In addition, there might be regional differences in the tolerability of capecitabine with gastrointestinal toxicities being worse in the US population compared with rest of the world (Haller *et al*, 2008; Hochster *et al*, 2008). Herein, we report our experience in CAPOX-associated toxicity before and after the introduction of our institutional policy to decrease capecitabine dose from 2000 mg m^–2^ per day (CAPOX 2000) to 1700 mg m^–2^ per day (CAPOX 1700) when combined with oxaliplatin in the treatment of CRC.

## PATIENTS AND METHODS

This analysis was approved by the Royal Marsden Hospital Insitutional Review Board (reference number: GI 107). To be eligible for inclusion into this study, patients were ⩾18 years of age, had histological diagnosis of colorectal adenocarcinoma and treated with CAPOX in either (neo)adjuvant or advanced disease settings with a minimum of one cycle of chemotherapy. The CAPOX was given as first line treatment for all patients with advanced disease. During the study period, patients were treated with neoadjuvant CAPOX in two prospective studies in MRI-defined high-risk localised rectal cancer (Chau *et al*, 2006; Chua *et al*, 2010). In addition, patients with stage III CRC were treated with adjuvant CAPOX both within (Haller *et al*, 2010) or outside clinical trials.

Following the reporting of high rates of grades 3 and 4 diarrhoea and dehydration with CAPOX 2000 in the TREE-1 study (Hochster *et al*, 2008) and the initial observation of higher grades 3 and 4 diarrhoea in our prospective EXPERT-C study, we instituted a dose reduction of capecitabine in August 2006 from CAPOX 2000 to CAPOX 1700. In addition, patients aged ⩾75 were recommended to have reduced starting dose of 1500 mg m^–2^ per day for CAPOX 2000 and 1300 mg m^–2^ per day for CAPOX 1700.

Patients were identified from our hospital electronic patient record (EPR) and pharmacy database, which comprised all patients who receive treatment at the Royal Marsden Hospital. The first 200 patients treated with CAPOX before and 200 patients after our institutional dose change were selected. In our institution, patients with significant pre-existing neuropathy or renal impairment (creatinine clearance <30 ml min^–1^) would not receive CAPOX for CRC.

Details of patients’ demographics, starting dose, dose reduction and cessation of CAPOX; toxicity including neutropenia, thrombocytopenia, diarrhoea, skin rash, hypersensitivity, lethargy, hand foot syndrome (HFS), stomatitis, peripheral neuropathy, constipation, abdominal pain/cramps, chest pain/cardiac events; hospitalisation relating to chemotherapy-induced toxicity and deaths during hospitalisation were collected retrospectively. An estimation of patients’ baseline renal function (estimated glomerular filtration rate (eGFR)) was performed using serum creatinine, patient age, gender and race according to the method described by Levey *et al* (1999). Serum bilirubin, alanine transferase (ALT) and alkaline phosphatase during CAPOX treatment were also collected. However, liver dysfunction might not necessarily be due to CAPOX treatment, as patients might have baseline dysfunction due to underlying liver metastases or secondary to concomitant medications such as antibiotics. All case notes and investigation results from all patients were available in our hospital EPR system. Discrepancies were verified with patients’ written notes if necessary and the database were appended accordingly. All toxicities were assessed according to the National Cancer Institute Common Terminology Criteria for Adverse Events version 3.

The primary aim of our study was to compare toxicity, in particular diarrhoea, in patients receiving CAPOX in our institution before (pre-2006 CAPOX 2000) and after (post-2006 CAPOX 1700) the dose change in August 2006. Other outcomes of interests were toxicity-related hospital admissions and proportions of patients requiring dose reduction or early termination of treatment.

With 200 patients in each group (400 patients in total), we could detect a decrease in incidence of grades 3 and 4 diarrhoea from 20% in the pre-2006 CAPOX 2000 group to 10% in the post-2006 CAPOX 1700 group with 80% power, two-sided *α*=0.05. Proportions of patients were compared using Fisher's exact test. Mean length of hospital stay was compared between the two groups using paired *t*-test. We performed univariate and multivariate logistic regression analyses to assess the impact of treatment groups (CAPOX 2000 *vs* CAPOX 1700) and other clinical determinants on incidence of toxicities (diarrhoea, stomatitis, HFS, neutropenia, elevated bilirubin, ALT and alkaline phosphatase). Apart from treatment groups, other clinical factors included were disease extent (metastatic *vs* adjuvant), gender (male *vs* female), race (white *vs* non-white), age (<70 *vs* ⩾70) and renal function (eGFR >70 *vs* 50–70 *vs* <50 ml min^1^). The first factor in each of these categories and CAPOX 2000 were used as control and their risk ratios (RR) set at 1. RR >1 indicated higher incidence of toxicities. Factors with *P*<0.05 in the univariate analysis were entered into the multivariate model.

## RESULTS

Between May 2005 and November 2008, 400 patients were included in this analysis with 200 in the CAPOX 2000 group and 200 in the CAPOX 1700 group. Table 1 shows baseline characteristics for patients included in this study. Thirty and 37 patients were aged over 75 in the CAPOX 2000 and CAPOX 1700 groups, respectively, and, therefore, had a reduced starting dose. Table 2 shows the toxicities encountered during CAPOX treatment. There were no significant differences between CAPOX 2000 and CAPOX 1700 for grades 3 and 4 diarrhoea (41 out of 200 *vs* 38 out of 200; *P*=0.80) and all grades diarrhoea (117 out of 200 *vs* 102 out of 200; *P*=0.16). There were also no significant differences for grades 3 and 4 stomatitis (0 out of 200 *vs* 2 out of 200; *P*=0.50), all grades stomatitis (27 out of 200 *vs* 29 out of 200; *P*=0.89) or grades 2–4 HFS (31 out of 200 *vs* 21 out of 200; *P*=0.18). Grades 3 and 4 as well as all grades neutropenia were significantly reduced with CAPOX 1700 (*P*=0.02 and 0.03, respectively) compared with CAPOX 2000. Furthermore, CAPOX 1700 was associated with significantly reduced incidence of all grades elevated bilirubin (*P*<0.0001) and ALT (*P*=0.0161). Grades 3 and 4 elevated bilirubin, ALT and alkaline phosphatase were infrequently encountered and were not significantly different between CAPOX 2000 and CAPOX 1700.

Although there was no significant difference in the rate of hospitalisation due to grades 3–4 toxicities (26 out of 200 *vs* 21 out of 200; *P*=0.53), for those patients requiring hospitalisation, CAPOX 1700 resulted in a longer mean length of hospital stay compared with CAPOX 2000 (paired *t*-test; *P*=0.0021). Table 3 shows these results. The number of cycles of CAPOX treatment was similar between the two groups with similar numbers of patients requiring dose reduction in first four cycles as well as those requiring to terminate treatment before completing four cycles of treatment.

Table 4 shows the results of the univariate and multivariate logistic regression analyses to assess the impact of treatment groups (CAPOX 2000 *vs* CAPOX 1700) and other clinical determinants on incidence of toxicities. The CAPOX 1700 was associated with significantly less neutropenia and liver dysfunction (elevated serum bilirubin and ALT) on univariate and multivariate analyses. Interestingly, (neo)adjuvant therapy was generally associated with higher incidence of toxicities (RR>1), although only statistically significant in elevated ALT. Similarly, females were associated with higher incidence of toxicities, significantly so in grades 3 and 4 diarrhoea. The only exception was all grades elevated bilirubin in which females experienced less toxicities. Mild renal impairment (eGFR: 50–70 ml min^–1^) was associated with significantly more severe diarrhoea, but not other toxicities.

## DISCUSSION

In this retrospective analysis of 400 patients, we did not detect any differences in the proportion of patients experiencing severe diarrhoea between CAPOX 2000 and CAPOX 1700 – a major reason that prompted dose reduction of capecitabine in other studies. Whereas our cohorts of patients were treated in two different time periods, the indications for our patients to receive CAPOX within our institution would be similar. This finding was a surprise to us as there was a general feeling that CAPOX 2000 was less tolerable.

One needs to take into account that our cohorts were derived from routine clinical practice, rather than from a prospective clinical trial population. The median age in our patient population was 65 years, comparable with 60–66 years old in other studies (Diaz-Rubio *et al*, 2007; Fuchs *et al*, 2007; Porschen *et al*, 2007; Hochster *et al*, 2008; Haller *et al*, 2010). Collection of toxicity data was through medical chart review and thus not as complete as prospective toxicity data collection. Nevertheless, it would be unusual for severe (grades 3 and 4) toxicity reporting to be omitted from the case notes. Similarly, although patients could have been hospitalised outside our institution, it would be our routine practice to admit patients into our institution for chemotherapy-related toxicities. Whereas data on dose reduction and early termination of treatment were collected in our study, we did not collect data on dose delay, as a variety of reasons could contribute to dose delay including patients’ wish or public holidays, rather than related to toxicity. As our study was conducted in real life situation, therefore, not to the stringency of prospective clinical trials, we could not report the dose intensities in either treatment group (CAPOX 2000 *vs* CAPOX 1700).

In randomised-controlled trials (RCTs), the rates of diarrhoea do not differ whether CAPOX was given in the adjuvant or metastatic disease setting (Schmoll *et al*, 2007; Cassidy *et al*, 2008; Rothenberg *et al*, 2008; Haller *et al*, 2010). Table 5 shows grades 3 and 4 toxicities encountered in RCTs evaluating CAPOX (Diaz-Rubio *et al*, 2007; Porschen *et al*, 2007; Cassidy *et al*, 2008; Rothenberg *et al*, 2008; Haller *et al*, 2010). With CAPOX 2000, the rates of grades 3 and 4 diarrhoea were about 20% (Cassidy *et al*, 2008; Rothenberg *et al*, 2008; Haller *et al*, 2010). In TREE-1 study, CAPOX 2000 was associated with grades 3 and 4 diarrhoea in 31% and dehydration in 27% of patients (Hochster *et al*, 2008). As a result, the dose of CAPOX was reduced to 1700 mg m^–2^ per day in the subsequent cohort when bevacizumab was added to CAPOX. The efficacy was not compromised with the reduced dose. An objective response rate of 27% and 46% and median overall survival of 17.2 and 24.6 months were seen with CAPOX 2000 and CAPOX 1700 plus bevacizumab, respectively. As no improvement in response rate or overall survival was observed when bevacizumab was added to CAPOX in a randomised study (Saltz *et al*, 2008), the numerically better response rate and overall survival seen in TREE-1 study in CAPOX 1700 plus bevacizumab could not be accounted for by the addition of bevacizumab. We did not assess efficacy end points in our study, as patients could be treated in either (neo)adjuvant or metastatic setting. Furthermore, the baseline prognostic factor could be variable, for example resectable metastatic liver only disease *vs* unresectable widespread metastatic disease.

Potential regional differences have been reported in the safety profiles for capecitabine (Haller *et al*, 2008). Grades 3 and 4 toxicities, in particular gastrointestinal toxicities, were significantly more frequent in the United States compared with non-US population. These toxicities led to significantly more treatment discontinuation. These findings were seen in both metastatic and adjuvant disease settings. In the adjuvant CAPOX study, NO16968, the main differences were between United States and East Asia with the Europeans having overlapping 95% confidence interval with the US population in the relative risk of treatment-related toxicities.

In another UK COIN study, significantly higher rates of grades 3 and 4 diarrhoea were reported when CAPOX 2000 was combined with cetuximab (17% CAPOX alone *vs* 30% CAPOX plus cetuximab; *P*<0.001) (Adams *et al*, 2009). As a result, the COIN Trial Management Group reduced CAPOX 2000 to CAPOX 1700 when combined with cetuximab. After dose reduction, the rates of grade 3+ diarrhoea decreased from 30% to 16%. The rates of grade 3+ diarrhoea for CAPOX 2000 without cetuximab in this COIN trial were 12–17%, as compared with 20% seen in our study for CAPOX 2000.

We also investigated the relationship between CAPOX dose and liver dysfunction. As liver dysfunction could be due to underlying liver metastasis, infection or concomitant medications, not all toxicities observed were treatment related. However, it was notable that in the original phase I dose-finding trial for CAPOX, there was a 33% grades 3 and 4 liver toxicity rate observed in CAPOX 2000, but there was no grades 3 and 4 liver toxicities encountered in the dose level below (CAPOX 1650) (Diaz-Rubio *et al*, 2002). In our study, CAPOX 1700 was associated with significantly reduced incidence of elevated bilirubin and ALT. However, most liver toxicities were of grades 1 and 2, fully reversible and did not require specific management.

With regard to other clinical factors, which might predict toxicities in our study, patients treated within the (neo)adjuvant setting had a general non-significant increase in toxicity in our univariate analysis. This might reflect clinicians’ reluctance for dose reduction in this setting, resulting in higher incidence of toxicities. The only exception was elevated serum alkaline phosphatase being more frequent in metastatic setting, which highlighted the underlying liver metastases in these patients. Females experienced more grades 3 and 4 diarrhoea in our study. Females have been found to experience greater fluorouracil toxicity than men with CRC (Sloan *et al*, 2002) in a meta-analysis of five RCTs in advanced and adjuvant settings. Women and their tumours may be more sensitive to 5-FU than men and lower doses of 5-FU may have an increased pharmacological and antitumour effect in females. It is well recognised that patients with moderate renal impairment (creatinine clearance 30–50 ml min^–1^) would experience a higher incidence of grade 3 or 4 toxicities (Cassidy *et al*, 2002). The phase III data and an additional pharmacokinetic study support a lower starting dose in these patients. This was indeed practised in our study patients, although very few (*n*=11) patients had eGFR <50 ml min^–1^. This might explain the favourable safety seen in this group of patients in our study. However, published data on mild renal impairment (50–70 ml min^–1^) are few. In our study, mild renal impairment was associated with higher frequency of severe diarrhoea. This was important as the coupling of diarrhoea with mild renal impairment could quickly lead to dehydration and acute renal failure. Thankfully no other toxicities were significantly increased in patients with mild renal impairment. Furthermore, the reduced starting dose for our elderly patients again led to favourable safety profile with no significantly increased toxicities compared with those younger than 70 years old.

In conclusion, in our cohort of 400 patients, we did not detect any statistically or clinically significant differences in GI toxicities or hospitalisation rates after reducing our routine capecitabine dose from CAPOX 2000 to CAPOX 1700. However, there was reduced frequency of neutropenia and liver dysfunction with the use of CAPOX 1700.

## Figures and Tables

**Table 1:** Baseline characteristics

	**Pre-2006 CAPOX 2000**	**Post-2006 CAPOX 1700**
N	200	200
Median age (years) (range)	65 (23–83)	66 (23–87)
Aged⩾75 years	30 (15%)^a^	37 (19%)^b^
Aged <75 years	170 (85%)	163 (82%)
		
*Gender*		
Male	118 (59%)	121 (61%)
Female	82 (41%)	79 (39%)
		
*Race*		
White	177 (89%)	186 (93%)
Asian	8 (4%)	11 (6%)
Black	11 (6%)	3 (2%)
Others	4 (2%)	0 (0%)
		
*Treatment intention*		
(Neo)adjuvant	68 (34%)	48 (24%)
Metastatic	132 (66%)	152 (76%)
		
*Baseline eGFR*		
Median (ml min^–1^) (range)	83 (31–151)	83 (40–214)
<50 ml min^–1^	6 (3%)	5 (3%)
50–70 ml min^–1^	44 (22%)	49 (25%)
>70 ml min^–1^	150 (75%)	146 (73%)

Abbreviations: CAPOX=capecitabine plus oxaliplatin; eGFR=estimated glomerular filtration rate.

This table shows number of patients in each category unless otherwise stated (i.e. median age and median eGFR).

a Aged ⩾75 years and had capecitabine starting dose of 1500 mg m^–2^ per day instead of 2000 mg m^–2^ per day.

b Aged ⩾75 years and had capecitabine starting dose of 1300 mg m^–2^ per day instead of 1700 mg m^–2^ per day.

**Table 2:** Toxicity and hospitalisation during CAPOX treatment

	**CAPOX 2000 (*n*=200)**				**CAPOX 1700 (*n*=200)**			
**Toxicity**	**G1**	**G2**	**G3**	**G4**	**G1**	**G2**	**G3**	**G4**
Diarrhoea	60 (30%)	16 (8%)	40 (20%)	1 (0.5%)	40 (20%)	24 (12%)	35 (17.5%)	3 (1.5%)
Lethargy	47 (23.5%)	11 (5.5%)	15 (7.5%)	0 (0%)	56 (28%)	34 (17%)	33 (16.5%)	1 (0.5%)
Hand foot syndrome	23 (11.5%)	18 (9%)	13 (6.5%)	0 (0%)	21 (10.5%)	8 (4%)	13 (6.5%)	0 (0%)
Stomatitis	22 (11%)	5 (2.5%)	0 (0%)	0 (0%)	22 (11%)	5 (2.5%)	2 (1%)	0 (0%)
Peripheral neuropathy	97 (48.5%)	16 (8%)	12 (6%)	0 (0%)	109 (54.5%)	24 (12%)	10 (5%)	0 (0%)
Chest pain^a^	0 (0%)	0 (0%)	6 (3%)	0 (0%)	4 (2%)	0 (0%)	4 (2%)	0 (0%)
Laboratory data								
Neutropenia	7 (3.5%)	12 (6%)	16 (8%)	3 (1.5%)	7 (3.5%)	8 (4%)	7 (3.5%)	0 (0%)
Elevated bilirubin	77 (38.5%)	37 (18.5%)	5 (2.5%)	0 (0%)	47 (23.5%)	27 (13.5%)	5 (2.5%)	0 (0%)
Elevated ALT	87 (43.5%)	16 (8%)	3 (1.5%)	0 (0%)	55 (27.5%)	21 (10.5%)	5 (2.5%)	0 (0%)
Elevated alkaline phosphatase	63 (31.5%)	12 (6%)	2 (1%)	0 (0%)	77 (38.5%)	11 (10.5%)	4 (2%)	0 (0%)

*Hospitalisation rate due to grades 3 out of 4 toxicities*								
Yes	26 (13%)				21 (10.5%)			
No	174 (87%)				179 (89.5%)			

*Length of hospitalisation*								
Mean	4.91 days				10.53 days			
s.d.	4.73 days				9.99 days			
Median	3 days				6.5 days			
Range	1–21 days				(1–39 days)			

*Deaths during hospital admission*	0				2			

Abbreviations: ALT=alanine transferase; CAPOX=capecitabine plus oxaliplatin; s.d.=standard deviation; G=grade.

a Considered to be cardiac in origin.

**Table 3:** Drug delivery during CAPOX treatment

	**CAPOX 2000**	**CAPOX 1700**
Median number of cycles (range)	7 (1–20)	5 (1–18)
No. of patients with dose reduction in first four cycles, but continue treatment	60 (30%)	57 (28.5%)
No. of patients who stopped treatment before cycle four CAPOX	32 (16%)	46 (21.5%)

Abbreviation: CAPOX=capecitabine plus oxaliplatin.

**Table 4:** Univariate and multivariate analyses of clinical determinants and toxicities

		**Univariate analyses**			**Multivariate analyses**		
**Clinical factors**	**No. of patients**	**Risk ratio**	**95% confidence interval**	***P***	**Risk ratio**	**95% confidence interval**	***P***
*Grades 3 and 4 diarrhoea*							
Treatment groups							
CAPOX 2000	200	1					
CAPOX 1700	200	0.88	0.53–1.45	0.61	NA		
Disease extent							
Metastatic	284	1					
(Neo)adjuvant	116	1.65	0.97–2.80	0.07	NA		
Gender							
Male	239	1			1		
Female	161	1.74	1.05–2.90	0.03	1.69	1.01–2.83	0.047
Race							
White	363	1					
Non-white	37	0.67	0.25–1.77	0.42	NA		
Age							
<70 years old	262	1					
⩾70 years old	138	0.77	0.44–1.32	0.34	NA		
Baseline eGFR							
>70 ml min^–1^	296	1			1		
50–70 ml min^–1^	93	2.34	1.36–4.03	0.002	2.27	1.31–3.92	0.003
<50 ml min^–1a^	11						
							
*All grades diarrhoea*							
Treatment groups							
CAPOX 2000	200	1					
CAPOX 1700	200	0.74	0.50–1.10	0.13	NA		
Disease extent							
Metastatic	284	1					
(Neo)adjuvant	116	1.31	0.85–2.03	0.23	NA		
Gender							
Male	239	1					
Female	161	1.23	0.82–1.84	0.32	NA		
Race							
White	363	1					
Non-white	37	0.41	0.20–0.84	0.01	NA		
Age							
<70 years old	262	1					
⩾70 years old	138	1.46	0.96–2.23	0.08	NA		
Baseline eGFR							
>70 ml min^–1^	296	1					
50–70 ml min^–1^	93	1.42	0.88–2.29	0.15	NA		
<50 ml min^–1^	11	1.08	0.32–3.61	0.90			
							
*All grades stomatitis*							
Treatment groups							
CAPOX 2000	200	1					
CAPOX 1700	200	1.09	0.62–1.91	0.77	NA		
Disease extent							
Metastatic	284	1					
(Neo)adjuvant	116	1.88	1.05–3.37	0.03	NA		
Gender							
Male	239	1					
Female	161	1.34	0.76–2.37	0.31	NA		
Race							
White	363	1					
Non-white	37	1.05	0.39–2.81	0.93	NA		
Age							
<70 years old	262	1					
⩾70 years old	138	0.73	0.39–1.35	0.32	NA		
Baseline eGFR							
>70 ml min^–1^	296	1					
50–70 ml min^–1^	93	0.60	0.28–1.28	0.18	NA		
<50 ml min^–1^	11	1.24	0.26–5.93	0.79			
							
*Grades 2–4 hand foot syndrome*							
Treatment groups							
CAPOX 2000	200	1					
CAPOX 1700	200	0.64	0.35–1.16	0.14	NA		
Disease extent							
Metastatic	284	1			1		
(Neo)adjuvant	116	2.62	1.44–4.74	0.002	2.52	1.39–4.59	0.002
Gender							
Male	239	1					
Female	161	1.01	0.56–1.82	0.98	NA		
Race							
White	363	1					
Non-white	37	0.50	0.22–1.16	0.11	NA		
Age							
<70 years old	262	1			1		
⩾70 years old	138	0.47	0.23–0.94	0.03	0.49	0.24–1.00	0.05
Baseline eGFR							
>70 ml min^–1^	296	1					
50–70 ml min^–1^	93	1.50	0.79–2.85	0.22	NA		
<50 ml min^–1a^	11						
							
*Grades 3–4 neutropenia*							
Treatment groups							
CAPOX 2000	200	1					
CAPOX 1700	200	0.35	0.14–0.84	0.02	NA		
Disease extent							
Metastatic	284	1					
(Neo)adjuvant	116	1.58	0.70–3.59	0.28	NA		
Gender							
Male	239	1					
Female	161	2.13	0.95–4.76	0.07	NA		
Race							
White	363	1					
Non-white	37	1.30	0.37–4.57	0.68	NA		
Age							
<70 years old	262	1					
⩾70 years old	138	0.68	0.28–1.67	0.40	NA		
Baseline eGFR							
>70 ml min^–1^	296	1					
50–70 ml min^–1^	93	0.74	0.27–2.03	0.56	NA		
<50 ml min^–1a^	11						
							
*All grades neutropenia*							
Treatment groups							
CAPOX 2000	200	1					
CAPOX 1700	200	0.53	0.30–0.93	0.03	NA		
Disease extent							
Metastatic	284	1					
(Neo)adjuvant	116	1.39	0.78–2.48	0.27	NA		
Gender							
Male	239	1					
Female	161	1.47	0.85–2.56	0.17	NA		
Race							
White	363	1					
Non-white	37	0.61	0.26–1.40	0.24	NA		
Age							
<70 years old	262	1					
⩾70 years old	138	0.79	0.43–1.42	0.43	NA		
Baseline eGFR							
>70 ml min^–1^	296	1					
50–70 ml min^–1^	93	0.69	0.34–1.40	0.31	NA		
<50 ml min^–1^	11	0.52	0.06–4.13	0.53			
							
*All grades elevated serum bilirubin*							
Treatment groups							
CAPOX 2000	200	1			1		
CAPOX 1700	200	0.44	0.29–0.66	<0.001	0.43	0.29–0.64	<0.001
Disease extent							
Metastatic	284	1					
(Neo)adjuvant	116	1.02	0.66–1.57	0.92	NA		
Gender							
Male	239	1			1		
Female	161	0.61	0.41–0.91	0.02	0.59	0.39–0.88	0.01
Race							
White	363	1					
Non-white	37	1.08	0.55–2.12	0.83	NA		
Age							
<70 years old	262	1					
⩾70 years old	138	0.92	0.61–1.39	0.68	NA		
Baseline eGFR							
>70 ml min^–1^	296	1					
50–70 ml min^–1^	93	1.48	0.91–2.32	0.12	NA		
<50 ml min^–1^	11	0.62	0.18–2.18	0.46			
							
*All grades elevated serum ALT*							
Treatment groups							
CAPOX 2000	200	1			1		
CAPOX 1700	200	0.60	0.40–0.89	0.01	0.62	0.41–0.93	0.02
Disease extent							
Metastatic	284	1			1		
(Neo)adjuvant	116	1.77	1.14–2.73	0.01	1.63	1.05–2.54	0.03
Gender							
Male	239	1					
Female	161	1.43	0.96–2.14	0.08	NA		
Race							
White	363	1					
Non-white	37	0.85	0.43–1.68	0.64	NA		
Age							
<70 years old	262	1			1		
⩾70 years old	138	0.58	0.38–0.88	0.01	0.58	0.38–0.90	0.01
Baseline eGFR							
>70 ml min^–1^	296	1					
50–70 ml min^–1^	93	0.91	0.57–1.46	0.70	NA		
<50 ml min^–1^	11	0.92	0.28–3.09	0.90			
							
*All grades elevated serum alkaline phosphatase*							
Treatment groups							
CAPOX 2000	200	1					
CAPOX 1700	200	1.35	0.91–2.01	0.14	NA		
Disease extent							
Metastatic	284	1					
(Neo)adjuvant	116	0.43	0.27–0.69	<0.001	NA		
Gender							
Male	239	1					
Female	161	1.23	0.82–1.84	0.32	NA		
Race							
White	363	1					
Non-white	37	0.81	0.41–1.63	0.56	NA		
Age							
<70 years old	262	1					
⩾70 years old	138	0.72	0.47–1.10	0.13	NA		
Baseline eGFR							
>70 ml min^–1^	296	1					
50–70 ml min^–1^	93	0.80	0.50–1.29	0.36	NA		
<50 ml min^–1^	11	2.32	0.66–8.08	0.19			

Abbreviations: ALT=alanine transferase; CAPOX=capecitabine plus oxaliplatin; eGFR=estimated glomerular filtration rate; NA=not applicable.

These factors were non-significant in univariate analyses and, therefore, not entered into multivariate regression models. When less than 2 factors was significant in univariate analyses, no multivariate analysis was performed.

a Baseline eGFR <50 ml min^–1^ had too few events in the category to perform meaningful statistical analyses.

**Table 5:** Grades 3 and 4 toxicities encountered with CAPOX in selected randomised-controlled trials

	**NO16968**	**NO16966**	**NO16967**	**AIO**	**TTD**	**TREE**
Disease setting	Adjuvant	1st line metastatic	2nd line metastatic	1st line metastatic	1st line metastatic	1st line metastatic
Country of origin	Global	Global	Global	Germany	Spain	USA
Number of patients treated with CAPOX	938	655	311	235	171	48
						
*Grades 3 and 4 toxicities*						
Diarrhoea	20%	20%	19%	15%	14%	31%
Nausea and vomiting	9%	10%	7%	13%	8%	38%
Stomatitis	0.6%	1%	<1%	1%	2%	NR
Neutropenia	9%	6%	5%	NR	7%	15%
Hand foot syndrome	6%	6%	4%	10%	2%	19%
Neurosensory	11%	4%	<1%	25%	18%	21%

Abbreviations: CAPOX=capecitabine plus oxaliplatin; NR=not reported.
